# Supplementary material for: Constitutive activity of the inhibitory G protein pathway mediated by non-visual opsin Opn7b reduces cFos activity in stress and fear circuits and modulates avoidance behavior
Source: Front Behav Neurosci. 2025 Jun 5;19:1540947. doi: 10.3389/fnbeh.2025.1540947 (PMC12176751; doi:10.3389/fnbeh.2025.1540947)
Supplement: Supplementary file 1 [file Data_Sheet_1.docx]

**
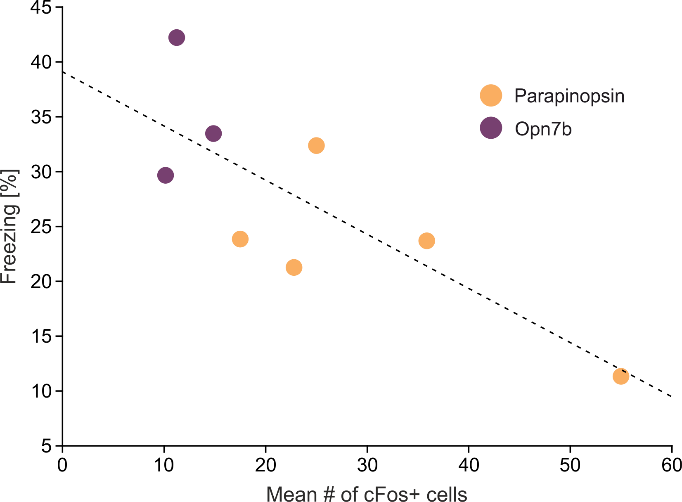
**

**Supplementary figure 1: Negative correlation between freezing behavior and VTA neuronal activity during the low 2MT condition.** Scatter plot showing a significant negative correlation (r = -0.80, p = .009) between the percentage of freezing during the low 2MT condition and the total number of cFos-positive cells in the ventral tegmental area (VTA). Each data point represents an individual subject. The line represents the linear regression fit.

| **Figure** | **Statistical Test** | **Independent Variable** | | **Dependent Variable** | | | | | | **n** | | | | | **t/U-Statistic** | | | | **df** | | | **z-value** | | | | **p-value** | **Cohen’s d/r-value** | | | |  |
| --- | --- | --- | --- | --- | --- | --- | --- | --- | --- | --- | --- | --- | --- | --- | --- | --- | --- | --- | --- | --- | --- | --- | --- | --- | --- | --- | --- | --- | --- | --- | --- |
| **1C** | Mann-Whitney U test | Treatment | | Total Distance moved | | Water | | | | | 12 | | 7.00 | | | | | - | | | -1.529 | | .154 | | | | | 0.44 | | | |
|  | Unpaired Student’s t-test |  |  |  |  | Low 2MT | | | | | 16 | | 0.23 | | | | | 14 | | | - | | .410 | | | | | 0.12 | | | |
|  |  |  |  |  |  | High 2MT | | | | | 16 | | 1.84 | | | | | 14 | | | - | | **.044** | | | | | **0.92** | | | |
| **1D** | Unpaired Student’s t-test | Treatment | | Mean Velocity | | Water | | | | | 12 | | 0.33 | | | | | 10 | | | - | | .374 | | | | | 0.20 | | | |
|  |  |  |  |  |  | Low 2MT | | | | | 16 | | 0.58 | | | | | 14 | | | - | | .285 | | | | | 0.29 | | | |
|  |  |  |  |  |  | High 2MT | | | | | 16 | | 0.23 | | | | | 14 | | | - | | .412 | | | | | 0.11 | | | |
| **1E** | Unpaired Student’s t-test | Treatment | | Time spent in Odor Zone | | Water | | | | | 12 | | -1.72 | | | | | 10 | | | - | | .058 | | | | | -1.05 | | | |
|  | Mann-Whitney U test |  |  |  |  | Low 2MT | | | | | 16 | | 7.00 | | | | | - | | | -2.626 | | **.007** | | | | | **0.66** | | | |
|  |  |  |  |  |  | High 2MT | | | | | 16 | | 15.00 | | | | | - | | | -1.788 | | .083 | | | | | 0.45 | | | |
| **1F** | Mann-Whitney U test | Treatment | | Freezing | | Water | | | | | 12 | | 28.00 | | | | | - | | | 2.075 | | **.048** | | | | | **0.60** | | | |
|  | Unpaired Student’s t-test |  |  |  |  | Low 2MT | | | | | 16 | | -0.25 | | | | | 14 | | | - | | .403 | | | | | - 0.13 | | | |
|  |  |  |  |  |  | High 2MT | | | | | 16 | | -1.48 | | | | | 14 | | | - | | .082 | | | | | -0.77 | | | |
| **1G** | Mann-Whitney U test | Treatment | | Number of cFos+ cells in BNST | | | | 162 | | | | | 1667.50 | | | | - | | | | -5.116 | | **< .001** | | | | | **0.40** | | | |
| **1H** | Mann-Whitney U test | Treatment | | Number of cFos+ cells in VTA | | | | 114 | | | | | 1736.50 | | | | - | | | | 0.997 | | .319 | | | | | 0.09 | | | |
| **2C** | Unpaired Student’s t-test | Treatment | | Total Distance moved | | | Water | | | 11 | | -0.79 | | | | | | 9 | | - | | | | | .226 | | | | -0.49 | | |
|  |  |  |  |  |  |  | Low 2MT | | | 14 | | 1.90 | | | | | | 12 | | - | | | | | **.041** | | | | **1.02** | | |
|  |  |  |  |  |  |  | High 2MT | | | 14 | | 3.20 | | | | | | 12 | | - | | | | | **.004** | | | | **1.71** | | |
| **2D** | Unpaired Student’s t-test | Treatment | | Mean Velocity | | | Water | | | 11 | | -0.58 | | | | | | 9 | | - | | | | | .289 | | | | -0.36 | | |
|  |  |  |  |  |  |  | Low 2MT | | | 14 | | -0.36 | | | | | | 12 | | - | | | | | .362 | | | | -0.19 | | |
|  |  |  |  |  |  |  | High 2MT | | | 14 | | -0.55 | | | | | | 12 | | - | | | | | .300 | | | | -0.29 | | |
| **2E** | Unpaired Student’s t-test | Treatment | | Time spent in Odor Zone | | | Water | | | 11 | | -3.13 | | | | | | 9 | | - | | | | | **.006** | | | | **-1.96** | | |
|  |  |  |  |  |  |  | Low 2MT | | | 14 | | 1.68 | | | | | | 12 | | - | | | | | .059 | | | | 0.90 | | |
|  | Mann-Whitney U test |  |  |  |  |  | High 2MT | | | 14 | | 29.00 | | | | | | - | | -3.071 | | | | | **.001** | | | | **0.82** | | |
| **2F** | Unpaired Student’s t-test | Treatment | | Freezing | | | Water | | | 11 | | 2.15 | | | | | | 9 | | - | | | | | **.030** | | | | **1.35** | | |
|  |  |  |  |  |  |  | Low 2MT | | | 14 | | -3.72 | | | | | | 12 | | - | | | | | **.001** | | | | **-1.99** | | |
|  |  |  |  |  |  |  | High 2MT | | | 14 | | -2.41 | | | | | | 12 | | - | | | | | **.016** | | | | **-1.29** | | |
| **2G** | Mann-Whitney U test | Treatment | | Number of cFos+ cells in VTA | | | | | 90 | | | | 168.00 | | | - | | | | | -6.472 | | **< .001** | | | | | | | **0.68** | |
| **2H** | Mann-Whitney U test | Treatment | | Number of cFos+ cells in BNST | | | | | 75 | | | | 470.00 | | | - | | | | | -2.145 | | **.032** | | | | | | | **0.25** | |
| **SF1** | Pearson Correlation | | Treatment | | Freezing | | Low 2MT | | | 8 | | | | - | | | 6 | | | | - | | | **.009** | | | | | | **-.80** |  |

**Supplementary table 1: Statistical procedures and results.**

Statistical comparison of parapinopsin or Opn7b injection (treatment) in the BNSTad **(1C-1H)** and VTA **(2C-2H)** regarding behavioural parameters and cFos expression.
